# Peptide libraries for comprehensive coverage of the tumor mutanome in immune monitoring and immunotherapy

**DOI:** 10.1186/2051-1426-3-S2-P5

**Published:** 2015-11-04

**Authors:** Aaron Castro, Maren Eckey, Tobias Knaute, Ulf Reimer

**Affiliations:** 1 JPT Innovative Peptide Technologies, Acton, MA, USA; 2 JPT Innovative Peptide Technologies, Berlin, Germany

## Background

Many cancers are associated with functional mutations which can be readily identified using new DNA sequencing methods. Sequence information is used for the generation of personalized cancer vaccines with promising clinical benefit. Intelligent ways to select the relevant, tumor-specific protein-coding mutations are applied to filter the large number of identified mutations to a manageable number. Besides, there is an increasing data pool of both germline and somatic mutations, for the latter frequently even with attached information on the tissue and histology. Although the use of a patient-specific antigen sequence represents a very promising basis for treatment and immune monitoring its identification and translation to corresponding reagents is still slow, laborious and expensive. Intelligently designed peptide libraries that cover sequence variability based on known antigen mutations may serve as easily accessible tools with broad exertion across patient cohorts in cases where the individual sequence is not readily available.

## Methods

Pools of antigen spanning peptides are increasingly used for T cell stimulation in T cell assays and even for immunotherapeutic treatment. Underlying peptide libraries are well suited for presenting sequence diversity. However, the currently used libraries consist of overlapping peptides of wild-type antigen sequences. We recently introduced a concept for diversity enhancement to the design of peptide pools covering immune relevant HIV antigens for which diversity between individual viruses poses many challenges. Exemplary, for the ENV protein an increase in sequence coverage from 10% to 34% could be achieved. In order to keep the number of individual peptides within peptide pools of tumor antigens at a controllable level, a complex filtering of sequence data is applied to enrich the library with peptides carrying different tumor-specific and ideally functionally relevant mutations.

Our aim is to generate peptide libraries for specific antigens, which represent a generic cancer mutanome applicable to a large number of individual patients, based on available databases as the catalogue of somatic mutations in cancer (COSMIC) and epitope information from the immune epitope database (IEDB) as well as prediction algorithms.

## Results

A library type can contain the full length sequence of the antigen enriched by peptides representing overlapping scans for relevant mutations. Alternatively, known or predicted epitopes can be selected and the mutated epitopes added. However, mutations can lead to the creation of new epitopes and mutated epitopes can escape from MHC-binding. Epitope prediction can help to account for these effects. We've built a pipeline for the efficient generation of such libraries which will be presented here.

